# The use of geochemical methods to pinpoint the origin of ancient white marbles

**DOI:** 10.1007/s00710-023-00833-2

**Published:** 2023-06-02

**Authors:** Walter Prochaska

**Affiliations:** Austrian Archaeological Institute/Austrian Academy of Sciences, Franz Klein-Gasse 1, A-1190 Vienna, Austria

**Keywords:** Marble provenance analysis, Stable isotope analysis, Trace element analysis, Inclusion fluid chemistry

## Abstract

“Multi-method-approach” has now been for many years the buzzword in marble provenance analysis. Nevertheless a true combination of the results of different analytical methods is rarely applied in the sense of the combined simultaneous use of a large number of analytically obtained numerical variables. It is demonstrated here that the combination of data from isotope analysis, chemical data, and data from the chemical analysis of inclusion fluids of an artefact and of course in combination with a corresponding database enhances substantially the accuracy of marble provenance analysis. It is explicitly pointed out that the unchallenged collection of data of the chemical composition of marbles from different sources (and different analytical procedures) most probably implies severe differences in their comparability. Exemplarily presented is the nearly perfect discrimination of the most important fine-grained marbles and furthermore the possibility of the intra-site discrimination of the three Carrara districts and the assignment of two portrait heads to the Carrara Torano quarries.

## Introduction

The earliest use of marble goes back to prehistoric times when pieces of jewellery e.g. pendants, idols, and also household goods like cups and pots were produced from this material. Later, from Archaic times on a marble industry developed on the Aegean islands famous for the production of kouroi and kore on the islands of Naxos, Paros and Thasos. The Greek east can look back on a long tradition of the use of marble in architecture, sculpture, and as a conveyor of information, while later, in the west, this phenomenon is connected with Roman influence. In the Roman period, a marked increase in the employment of marble can be observed in general, which is also understood as a typical example of the self-representation of the elites. Marble was specifically deployed in order to emphasise the socio-economic status of certain social groups. In the competition between elites, marble was assigned an important role due to its high prestige value, and can also be understood as an expression of political ties (Berns et al. 2002; Cancik et al. 2006).

**Fig. 1:**
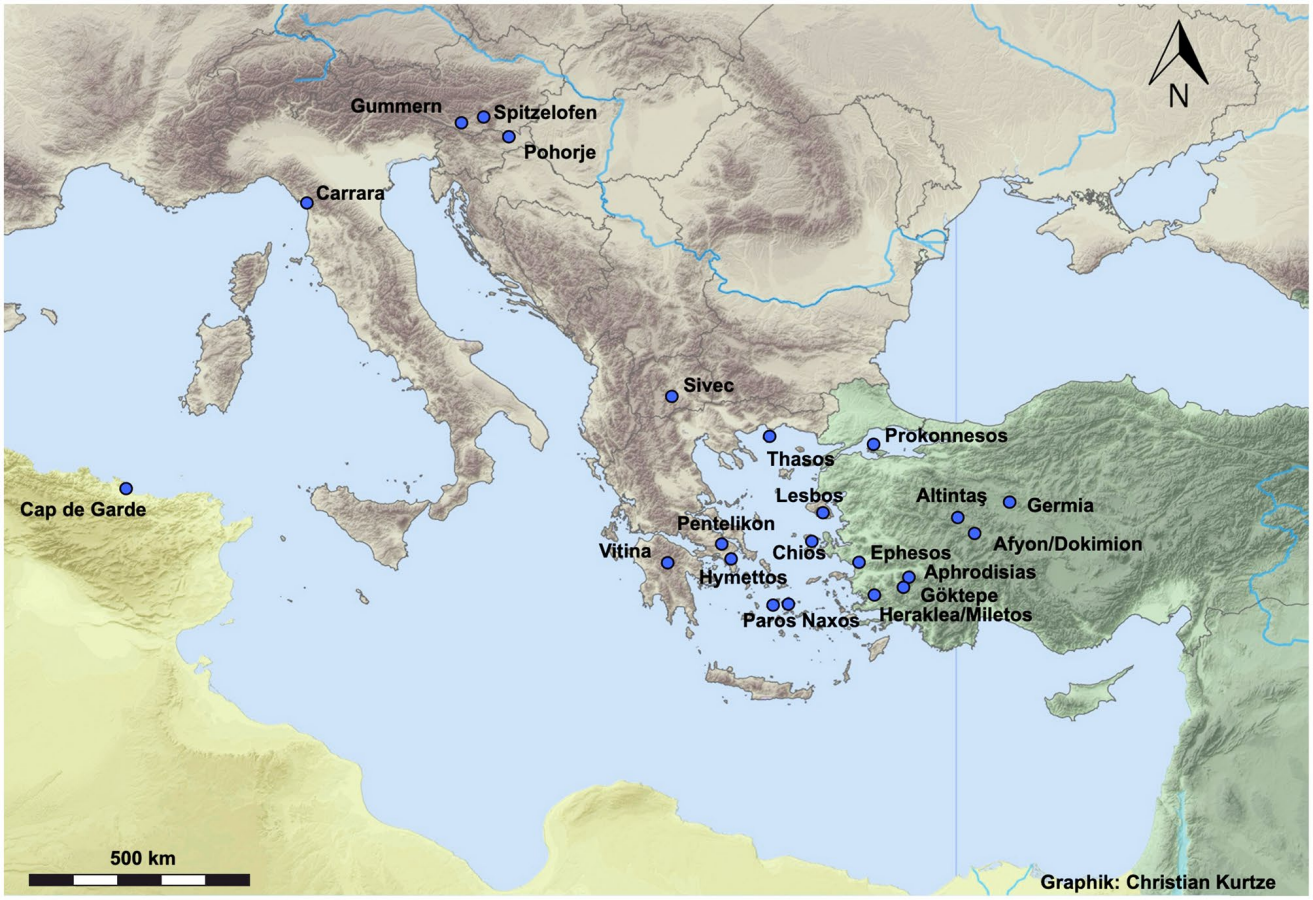
Map of the most important production sites of white marbles in antiquity (graphics by Christian Kurtze, Österreichisches Archäologisches Institut)

The increased level of urbanisation, associated with the foundation of new cities, and the development of already-existing ones, was connected with a great demand for building materials (Bowman and Wilson 2011). This expansion took place in differing geographical, chronological and even structural degrees and was heavily dependent on local conditions (Hanson 2016). The consequences were not only the opening up of new stone quarries, but also a direct correlation between stone quarries and cities. Many of the quarries known so far produced for a local market, often a regional one, but quite a large number of quarries is known that produced material for the international market traded over long distances (Fig. 1). This is equally the case for marble as well as for other types of stone. The above mentioned socio-political and cultural importance of marble attracts a great deal of attention of archaeologists and art historians interested in trading relations and cultural connections between different societies. Of special interest e.g. is the question whether the position of workshops is bound to a certain region or a special marble occurrence on one hand or - on the other hand - whether workshops migrated or travelling masters were active in different building projects.

To contribute to these investigations, methods had to be developed to assign the marble of an artefact to its source quarry or quarry region. In the case of the so-called coloured marbles the experienced eye is suffice to categorize the marble and pinpoint the source. Furthermore it is practically impossible to take small representative samples of these very heterogeneous marbles for scientific investigations.

Very much different is the characterization of white marbles for this purpose, where visual characterization with the aim to assign these marbles to their manifold sources is very limited. Therefore scientific methods have to be applied to differentiate between the white marbles of the many different source areas. As will be shown below so far no one single method was developed to safely distinguish between the different marbles on the base of one single methodological approach. Thus a combination of different analytical methods has to be applied to achieve a satisfactory separation of the corresponding compositional fields. Due to the large number of analytical variables acquired by this combination of different methods a statistical evaluation is indispensible.

This paper is based on the collection of a large set of quarry samples (approx. 4500 samples, November 2022) of white marbles from ancient quarries in the Greek and Roman world. Following the canons of science to grant reproducibility of a method, the access to the underlying data and their public availability, the database is continuously published (Prochaska and Attanasio 2021, 2022; Prochaska 2021) and available also on the website (https://www.walter-prochaska.at).

## The combination of different methods applied

### General remarks

The methods discussed below are those based on the above-mentioned database of approx. 4500 quarry samples, which were made publicly available by the author. In this databank the analytical results of each single sample for the three different analytical methods for these quarry samples are included. These methods are stable isotope analysis, trace element analysis and the analysis of the fluid inclusions of the marbles, and these methods will be discussed in more detail below. It is essential that the data are available in numerical figures, for any data expressed as language or in descriptive form cannot be included in the statistical evaluation procedure.

For assigning the marble of an artefact to its source the analytical results of this marble are computed together with the databank in a statistical evaluation. For the sake of comparison it is essential that the methods and analytical procedures for an artefact are exactly the same that were used for the samples of the databank. Analytical details and the exact procedures of the methodological conduct used are presented in Prochaska and Attanasio (2021, 2022) and Prochaska (2021).

In general a series of other analytical methods are used for the provenance analysis of ancient marbles, however, in these cases the data are often given as average data or in form of diagrams which cannot be used in concert with the data of our databank. For a simultaneous statistical evaluation it is imperative that all analytical data were carried out on the same quarry sample. The more important methods are mentioned below:

#### Electron paramagnetic resonance spectroscopy (EPR)  

was used very successfully, but during the last years it came out of use (e.g. Maniatis et al. 1988; Polykreti and Maniatis 2002; Attanasio 2003). The problem here is that the necessity of lab-internal standardisation prevents reproduction and the use of data obtained by other laboratories.

#### Cathodoluminescence

has been used for many years manly in a qualitative way and the resulting descriptive outcome is not suitable for a statistic evaluation together with other numerical data. However, recently a quantitative approach using cathodoluminescence was published and used with success (Blanc et al. 2020).

#### Grain-size

of the marbles is often expressed in MGS (maximum grain-size) and frequently used in marble provenance analysis. However, this parameter is here considered to be relatively uncertain because of the very small size of the samples that can usually be obtained from artefacts, and this by far does not meet petrographic standards. Therefore in many cases microscopic slides cannot be prepared and the visual evaluation with a hand magnifier is often not accurate.

### Stable isotope investigations

Craig and Craig (1972) published the first analyses on the stable isotope composition of O and C of ancient marbles on some quarries of the Greek islands and the mainland. In direct consequence this approach became the state of the art in this field of marble investigation. Since then a countless number of ancient marble samples were isotopically analysed during the last decades. Although an innumerable number of data was produced, and published collections or a comprehensive databank that are generally available are rare (e.g. Herz 1987; Gorgoni et al. 2002; Antonelli and Lazzarini 2015). Most of these published results are available as diagrams and not as numerical data thus these data cannot be used and combined with further analytical variables and therefore are of limited benefit for further users. The largest data collection with individual numerical results for each sample which is publicly available, was published in Attanasio et al. (2006) containing more that 1300 quarry samples. As the original samples of Attanasio et al. 2006 were available for this work for additional analyses (trace elements, fluid inclusion chemistry) the corresponding isotope numbers were used. Further isotope analyses were done in the laboratories of the University of Leoben using a Thermo Fisher Delta V mass spectrometer with a Finnigan Gas Bench II and a CTC Combi-Pal autosampler. The procedure followed the operation guidelines of Spötl and Vennemann (2003). Carbon isotope data and oxygen isotopes are reported relative to Vienna Pee Dee Belemnite (VPDB). Multiple measurements of in-house calcite reference material were used and precision of δ^18^O and δ^13^C measurement was at ± 0.07 ‰ and ± 0.05 ‰ respectively.

### Trace element analyses

In the 1960s, much earlier than the introduction of the stable isotope analysis for investigating the origin of ancient marbles, automated methods were introduced for chemical analyses. Different spectrographic methods like X-ray fluorescence (XRF), neutron activation (NAA) or inductively coupled plasma optical emission spectrometry (ICP-OES) were introduced in geochemical investigations. This led a number of geochemists to use trace element analyses for the characterization and differentiation of ancient marbles (e.g. Rybach and Nissen 1965; Conforto et al. 1975; Germann et al. 1980; Lazzarini et al. 1980; Oddone et al. 1985; Grimanis and Vassilaki-Grimani 1988; Mello et al. 1988; Moens et al. 1992; Cramer 2004; Poretti et al. 2017). However, this analytical approach was not pursued consequently and this method was considered to produce highly scattering data, which could not easily be compared with quarry data. Again a systematic collection of analytical data was not compiled.

One reason for the scarce success of these early geochemical analyses of very pure white marbles most probably was due to the very different methods applied for the chemical analyses which, by no means, must be mixed up in a data evaluation. Bulk analysis by NAA or XRF for instance must not be mixed up with near total dissolution by acid attack of the samples. In Cramer (2004) these issues are discussed in detail. This difference between a bulk analysis which also includes the silicates and insoluble accessory minerals (where e.g. the REE are concentrated) and the selective dissolution of the carbonate phase only becomes even much more important when considering the small amount of samples usually available.

Trace element analysis in this work was done on sample powders after a thorough cleaning of the chip samples. An amount of 0,1 g of fine ground powder was dissolved with concentrated hot HNO_3_ for about 5 min. As stated above it is of crucial importance to note that applying this procedure silicate contaminants in the marble are not dissolved, but only the carbonate phase and, if present, soluble minerals. The analyses were performed with an Agilent 8800 ICP Triple Quad (ICP-QQQ) mass spectrometer against the Merck VI standard. The running internal standard was limestone JLs-1.

### Fluid inclusion analysis

So-called “fluid inclusions” can be found practically in all kinds of crystalline rocks and minerals. For decades the study of the inclusion fluids was used in geological sciences and the number of papers published on this topic are legion. For several years now this method was also used for the provenance analysis of white marbles and supplies valuable proxies to achieve more precise results (e.g. Prochaska and Attanasio 2012; Prochaska and Grillo 2010). The inclusions represent the relics of the fluid/gaseous phase trapped during the crystallization or recrystallization of the corresponding minerals. The micro-cavities hosting these one or polyphase fluids rarely exceed few µm in size. When breaking up these inclusions in some types of marbles (e.g. the Prokonnesian marbles from the Marmara Island) emit a typical fetid odour due to the H_2_S content of the gaseous phase. The liquid phase exhibits different degrees of salinity and in the case of higher salinities may contain daughter crystals. The chemical composition of these inclusion fluids may vary in the different deposits and depends on the depositional environment of the carbonate sediment as well as on geologic processes that occurred during or after the lithification of the rock. On the scale of an outcrop or a quarry the general composition of the fluid does not vary much due to the homogenisation during metamorphism and recrystallization.

To extract the micro-inclusions in the marble samples, 1 g of cleaned sample was hand crushed using an agate mortar and pestle under 5 ml Milli-Q^®^ water. The resulting suspension was filtered with a syringe filter (nylon, < 0,2 μm) to separate the leachate from the ground powder. In special cases, smaller amounts of sample of approximately 0,2 g may be sufficient. A Dionex ion-chromatography system (DX-3000) was used for analysis of the anions. To obtain a minimum noise in the signal, external suppression was used. F^−^, Cl^−^, Br^−^, NO^3−^, SO_4_^2−^ and PO_4_^3−^ were analysed in one run. Li^+^, Na^+^, K^+^, Mg^2+^ and Ca^2+^ were analysed by a Dionex DX-120 ion-chromatography system with electrochemical auto-suppression. Because of the generally low concentrations of iodide in the inclusion fluids of the alabaster, I^−^ was analysed in a separate run using amperometric detection. An absolute prerequisite of the analytical equipment and the procedure is when analysing the extracted fluids very low detection limits for Br^−^ (~ 1 ppb) and for I^−^ (~ 0,1 ppb) can be obtained. Accuracy was tested by running standards and turned out to be at 5% for element ratios normalized to Na.

## Examples for the discrimination of ancient white marbles

### General remarks

Isotope analysis is the standard method to pinpoint white marbles to their origin. It is general opinion that no single analytical method will suffice to safely distinguish between the numerous quarries and mining sites where marble was extracted in antiquity. Nevertheless still solely isotope analyses are used with a backup of microscopic information, however, the result then are often ambiguous. Therefore the combination of the above-mentioned methods is used in the following. By means of two case studies it will be demonstrated below how the discrimination of marbles from different locations can be improved when using successively more analytical results and statistical discrimination analysis. The large number of acquired data invariably asks for the use of a statistical evaluation of the data. In this work the programme packages STATISTICA and SPSS were used.

### The example of distinguishing between fine-grained ancient white marbles

**Fig. 2:**
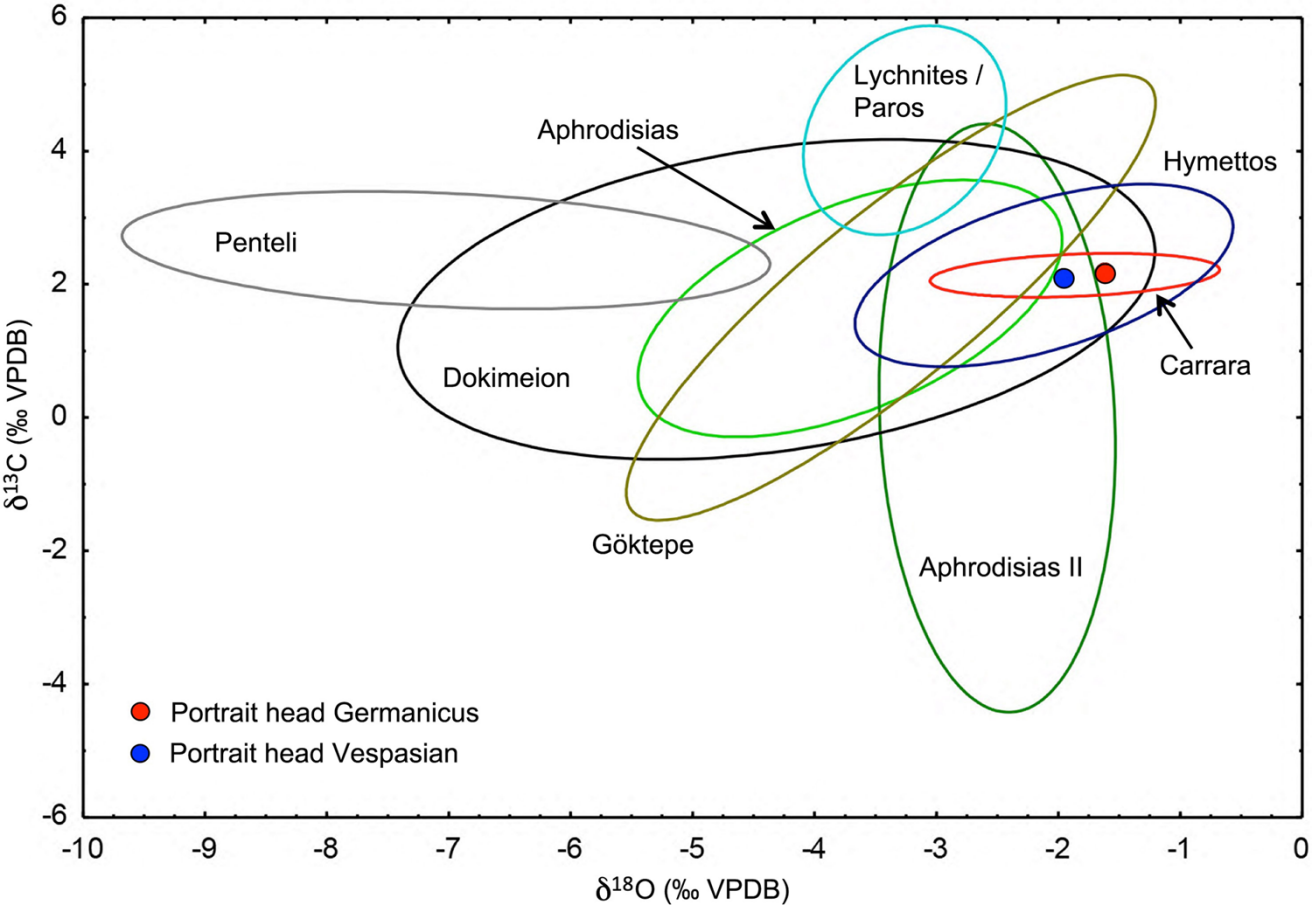
In the stable isotope diagram the different types of fine-grained marbles overlap considerably. The projection points of 2 artefacts discussed in the chapter below are also on display

The state of the art of provenance analysis in general is Stable Isotope analysis sometimes backed up by petrographic observations. However, the latter results are descriptive and cannot be included in a statistical evaluation. The discrimination of a series of most important white marbles of fine grain-size used in antiquity is shown exemplary below. The numerical data underlying these calculations are given in Prochaska and Attanasio (2022). These marbles were prominently used for architectural purposes, and due to their fine grain-size they play an outstanding role in art and sculpture. It will be demonstrated that a save separation of the marble sources can be achieved by using a combination of different analytical methods and subsequent statistical evaluation. Presented in the following are the marbles of fine grain-sized, most prominent and widely used in antiquity: Aphrodisias, Dokimeion, Penteli, Carrara, Göktepe and the Parian Lychnites. As is demonstrated in the isotope diagram in Fig. 2, there is a massive overlap of the corresponding compositional fields when using only stable isotope data. Thus discrimination solely on this basis is not possible.

The separation of the different data-fields can be improved substantially when applying a large number of variables with subsequent statistical discrimination analysis and consequently a good separation of the different marble sites can be achieved. In Table 1 the improvement of discrimination is shown by stepwise adding analytical variables obtained by different methods. Finally the best separation was achieved by using the variables ^18^O ‰, ^13^C ‰, Mn, Fe, Cr, Sr, Y, La, Ce, Pr, Yb, U, DS, Li/Na, Cl/Na, K/Na, Br/Na and I/Na. The corresponding calculated data (percentage of correct re-assignment of the quarry samples expressed in bold numbers) are presented in Table 1.

**Table 1:** Percentage of the correct re-assignments of the database samples

$$\mathrm{\delta^{18}O\;\%_{o},\,\delta^{13}C\;\%_{o}}$$								
	Aphrodisias I	Aphrodisias II	Dokimeion	Penteli	Carrara	Göktepe	Lychnites	Hymettos
Aphrodisias I	**19.5**	9.1	16.9	1.3	–	53.2	–	–
Aphrodisias II	–	**71.4**	–	–	14.3	–	–	14.3
Dokimeion	9.1	7.6	**27.3**	15.2	–	33.3	1.5	6.1
Penteli	–	–	19.4	**80.5**	–	–	–	–
Carrara	–	–	–	–	**65.2**	6.1	–	28.8
Göktepe	4.5	–	–	–	–	**92.9**	1.8	**–**
Lychnites	–	–	2.7	–	–-	2.7	**94.6**	**–**
Hymettos	–	4.2	4.2	–	41.7	**–**	**–**	**37.5**

$$\mathrm{\delta^{18}O\;\%_{o},\,\delta^{13}C\;\%_{o}},\,\mathrm{Mn,\; Fe\;Cr,\;Sr,\;Y,\;La,\;Ce,\;Pr,\;Yb,\;U}$$								
	Aphrodisias I	Aphrodisias II	Dokimeion	Penteli	Carrara	Göktepe	Lychnites	Hymettos
Aphrodisias I	**84.4**	1.3	9.1	1.3	1.3	2.6	–	–
Aphrodisias II	–	**85.7**	–	–	14.3	–	–	–
Dokimeion	4.5	–	**75.8**	15.2	3	1.5	–	–
Penteli	–	–	8	**89.7**	–	–	2.3	–
Carrara	7.6	–	–	–	**92.4**	–	–	–
Göktepe	12.5	–	1.8	–	–	**85.7**	–	**–**
Lychnites	–	–	–	–	–-	4.5	**94.6**	**–**
Hymettos	–	–	–	–	8.3	**–**	**–**	**91.7**

$$\mathrm{\delta^{18}O\;\%_{o},\,\delta^{13}C\;\%_{o}},\,\mathrm{Mn,\; Fe\;Cr,\;Sr,\;Y,\;La,\;Ce,\;Pr,\;Yb,\;U,\;Ds,\;K/Na, and\; I/Na}$$								
	Aphrodisias I	Aphrodisias II	Dokimeion	Penteli	Carrara	Göktepe	Lychnites	Hymettos
Aphrodisias I	**90.9**	–	2.6	2.6	–	2.6	1.3	–
Aphrodisias II	–	**100**	–	–	–	–	–	–
Dokimeion	4.5	–	**84.8**	7.6	1.5	1.5	–	–
Penteli	–	–	1.1	**98.9**	–	–	–	–
Carrara	1.5	–	–	–	**98.5**	–	–	–
Göktepe	4.5	–	–	–	–	**92.9**	1.8	**–**
Lychnites	–	–	–	–	–-	2.7	**97.3**	**–**
Hymettos	–	–	–	–	–	**–**	**–**	**100**

### The example of intra-site discrimination of the Carrara marbles

Having this very powerful tool at hand to separate marbles of different origin it is worth trying to advance into intra-site discrimination, that is to try to assign marbles of a prominent large marble site to their specific origin within this region. The famous Carrara marbles form Jurassic formations in the Northwest of the Italian Apuan Alps were exemplary selected for this purpose and are discussed below.

In the region of Carrara occasional marble quarrying took place possibly in pre-classical times, however, regular mining stated in Roman Republican times. In Roman Imperial times Carrara marble soon became the sculptural marble per se. From the early second century on Prokonnesian marble and Göktepe marble (exclusive sculptural marble) eroded this leading position of the Carrara marbles. Nevertheless continuous extensive mining throughout history until modern times caused an extensive destruction of the ancient mining traces. The Carrara quarries occur over a vast distance comprising three valleys or basins called Misegila, Colonnata and Torano and present-days activities take place in all three valleys. Several different qualities are produced presently, irregularly occurring all over the region in different abundance. The most famous type is the “statuario”, a highly esteemed flawless white variety occurring prevalently in the Torano basin.

**Fig. 3:**
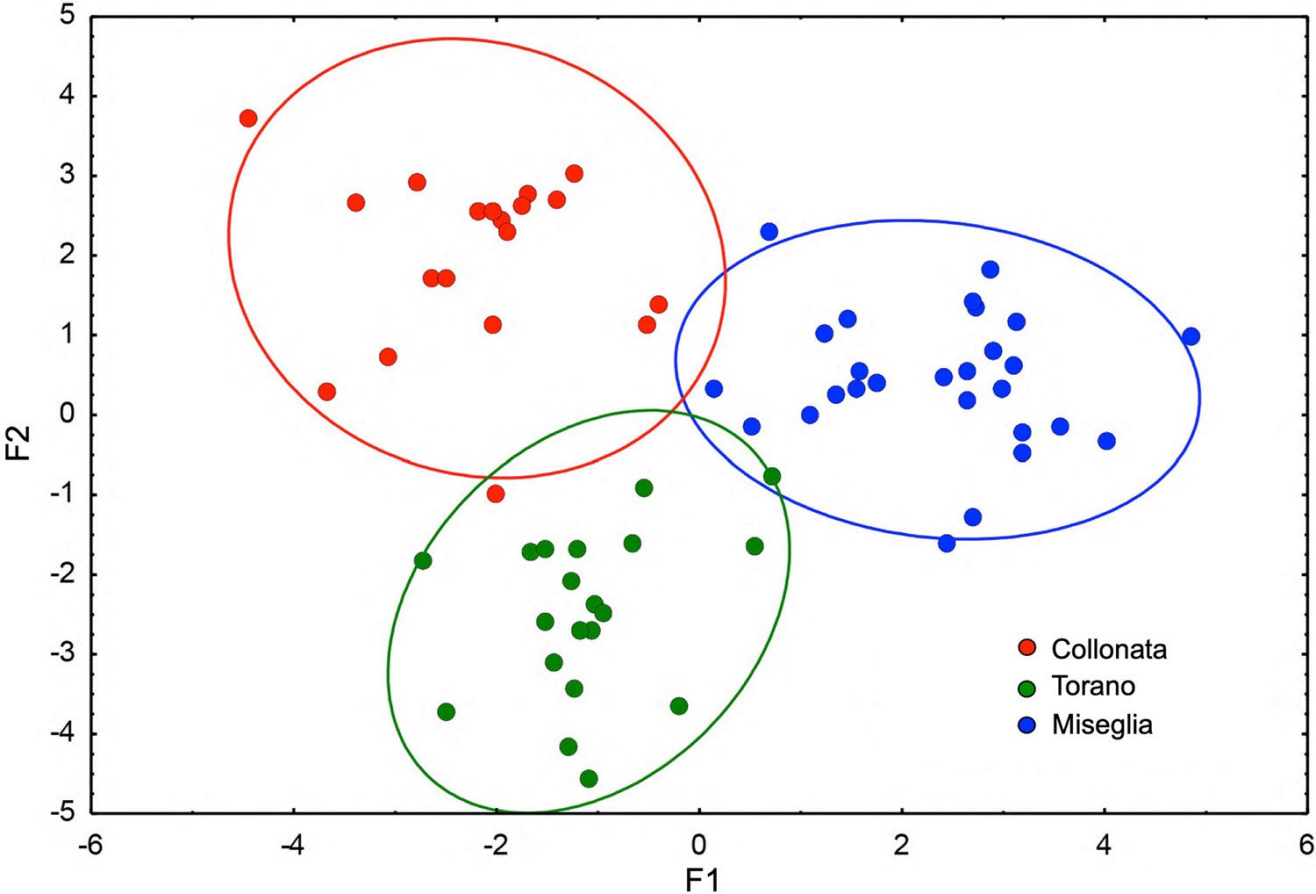
Bivariate diagram of the results of the multivariate discrimination of the three Carrara mining districts (Prochaska and Attanasio 2022)

So far it proved impossible to discriminate the marbles of the three valleys on the bases of isotope analysis. In 1986 Herz and Dean (1986) stated that “discriminant analysis (DA) of the isotopic analyses showed that the three quarry areas of Carrara could not be told apart…”. Attanasio et al. (2006) arrived at the same conclusion but got a slightly better result when computing EPR data together with the isotope results. However, Prochaska and Attanasio (2022) showed by combination of the three different methods discussed here that an almost perfect separation of the marbles of the three mining districts of Carrara is be achieved (Fig. 3).

**Fig. 4:**
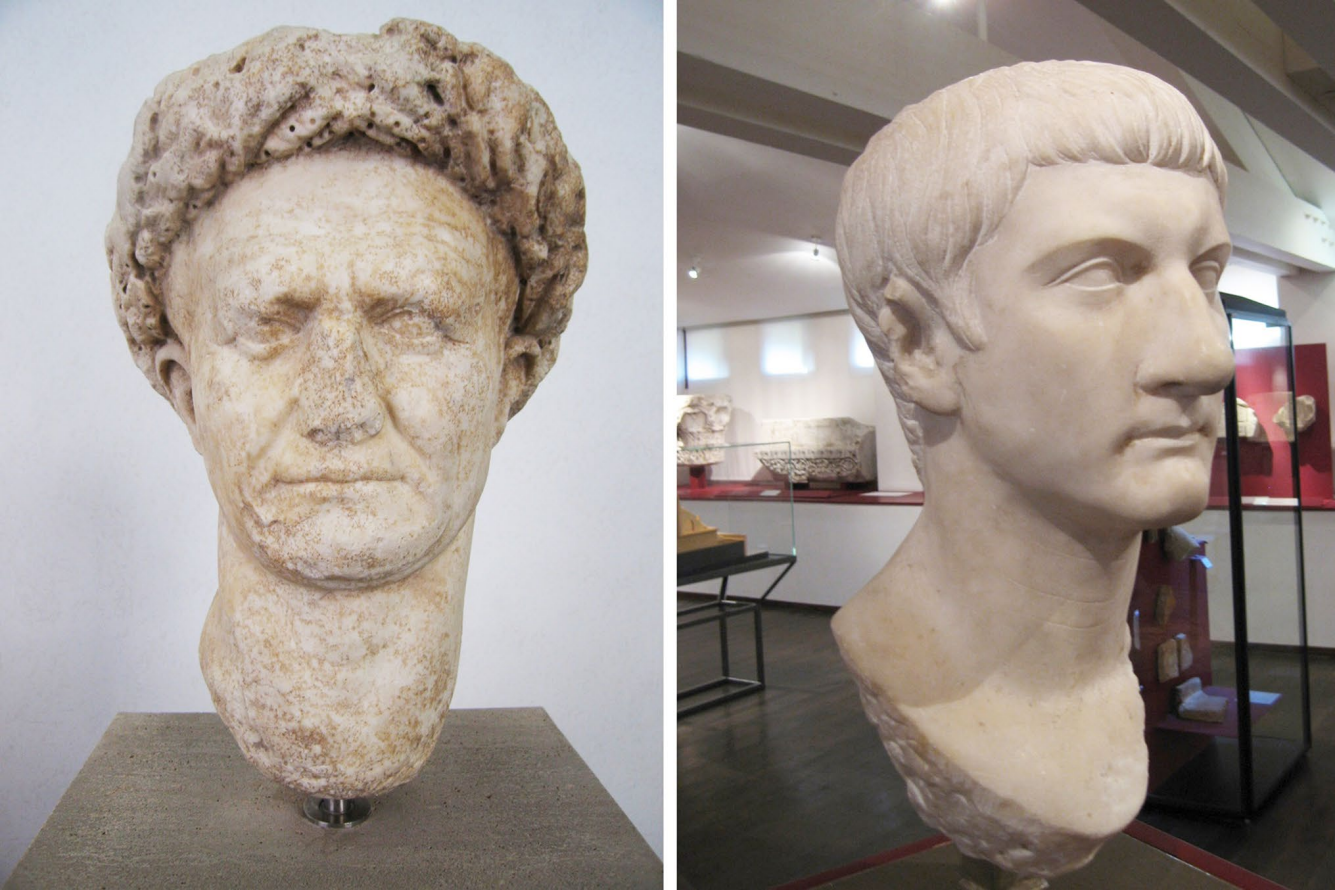
Two portraits made of Carrara marble discussed in the text. The portrait of Vespasian is from the Museo Nazionale Palazzo Massimo in Rome (inventory number 128571) and the head of Germanicus is from the Musée Saint-Raymond in Toulouse (inventory number 30010)

In the following an application of this possibility of intra-site discrimination will be shown based on the examples of two Imperial Roman portrait heads, one of Vespasian and the other of Germanicus (see Fig. 4). It was shown in previous investigations (Attanasio et al. 2019) that the marbles of the two portrait heads are clearly Carrara marbles. According to the above mentioned intra-site discrimination of the three Carrara districts the results of the two portrait heads were computed using the variables ^18^O ‰, ^13^C ‰, Mn, Fe, V, Y, La, Ce, Pr and Yb. The size of the samples of these two portrait heads was too small to also perform fluid analysis, nevertheless the results presented here for the provenance are quite clear and are presented in Table 2.

**Table 2:** Results of the statistical computing of the analytical results of the two analysed portrait heads using the variables ^18^O ‰, ^13^C ‰, Mn, Fe, V, Y, La, Ce, Pr and Yb

	Absolute probability %	Relative Probability %	Provenance	Relative Probability %	Provenance
	1^st^ choice			2^nd^ choice	
Vespasian	82.4	63.6	Torano	21.2	Colonnata
Germanicus	96.3	70.7	Torano	18.4	Miseglia

The relative probability is the probability of the sample to belong to one group of the groups selected for the calculation. The threshold is 60%. Lower values indicate that the sample’s assignment is in doubt between two or more groups. It is important that the 2^nd^ choice is substantially lower that the 1^st^ choice otherwise a safe assignment between the two choices is not possible. The relative probabilities of all groups under consideration sums up to 100%.

The absolute (typical) probability is a distance dependent parameter measuring the probability that the sample belongs to the chosen group or, in other words, is a typical representative of this specific group. The threshold is 10%, corresponding to samples on the edge of the 90% probability ellipse. Low values indicate anomalous samples (outliers) or samples possibly not belonging to any group in the selection.

## Discussion and conclusions

A safe discrimination of ancient white marbles can only be achieved by a combination of different methods as one method alone does not achieve sufficient selectivity. The large number of analytical variables obtained by different methods requires a statistical evaluation. Therefore only numerical results can be used for a combined assessment of all acquired outcomes. In this work the results of isotope analysis, trace element analysis and the analysis of the inclusion fluids were used for evaluation. The analytical results of the database are published as mentioned and specified above (see “Introduction”). The combined evaluation of the results is performed by discrimination analysis using the programme packages STATISTIKA and SPSS.

It is of paramount importance to apply exactly the same methods of investigation on the artefacts that had been used for establishing the databank. This is especially the case when using trace element analysis in the evaluation. High-quality pure white marble contains extremely low amounts of impurities and consists exclusively of calcite. The calcite lattice can incorporate Sr, Mn, Mg, and Fe in different amounts, but further trace elements are present in the very low of sub ppm level. On no account must bulk analyses like XRF or NAA be mixed up with analyses after partial dissolution of a sample (e.g. with HNO_3_), because in the latter case only the concentrations of the elements of the carbonate phase are recorded, while accessory minerals will not be dissolved and this would result in a deficiency in the findings of several trace elements, especially the REE. A key criterion for the use of a certain analytical method for the use in marble provenance analysis is the applicability when dealing with artefacts. Even when carefully selecting the spots where to take a sample, e.g. on the bottom of a base or on hidden surfaces, the acquired samples are usually very small. The required minimum sample after cleaning is 0,2 to 0,5 g to conduct all the methods proposed here. For a serious investigation of MGS or quantitative mineral composition in the petrographic microscope thin-sections in 3 different directions from one sample are required. In the rare cases where a microscopic slide can be prepared the investigation with the petrographic microscope or by SEM may be used as backup.

In general a reliable assignment of a marble sample to its quarry source can be achieved using combination of methods. As shown above it is furthermore possible in many cases to discriminate between single quarries of a larger marble site thus it is possible to trace back an artefact to a single quarry even in a complex marble site (e.g. Carrara or Ephesos).

Certainly the use of further discriminative variables would enhance the discriminatory power and selectivity of the procedure. These include more trace elements or further fluid inclusion parameters analysed exactly under the conditions described here. A further variable could possibly be obtained from quantitative cathodoluminescence although these results may to some extent duplicate the Mn-values of the trace element analyses. It is obvious that an enlargement of the number of variables of the databank requires that the analyses of any new parameters have to be performed at the very same samples utilized for the original databank.
